# Supplementary material for: Industrial and agricultural chemicals exhibit antimicrobial activity against human gut bacteria in vitro
Source: Nat Microbiol. 2025 Nov 26;10(12):3107–21. doi: 10.1038/s41564-025-02182-6 (PMC12669032; doi:10.1038/s41564-025-02182-6)

Uncropped gel image from Extended Data Figure 5A

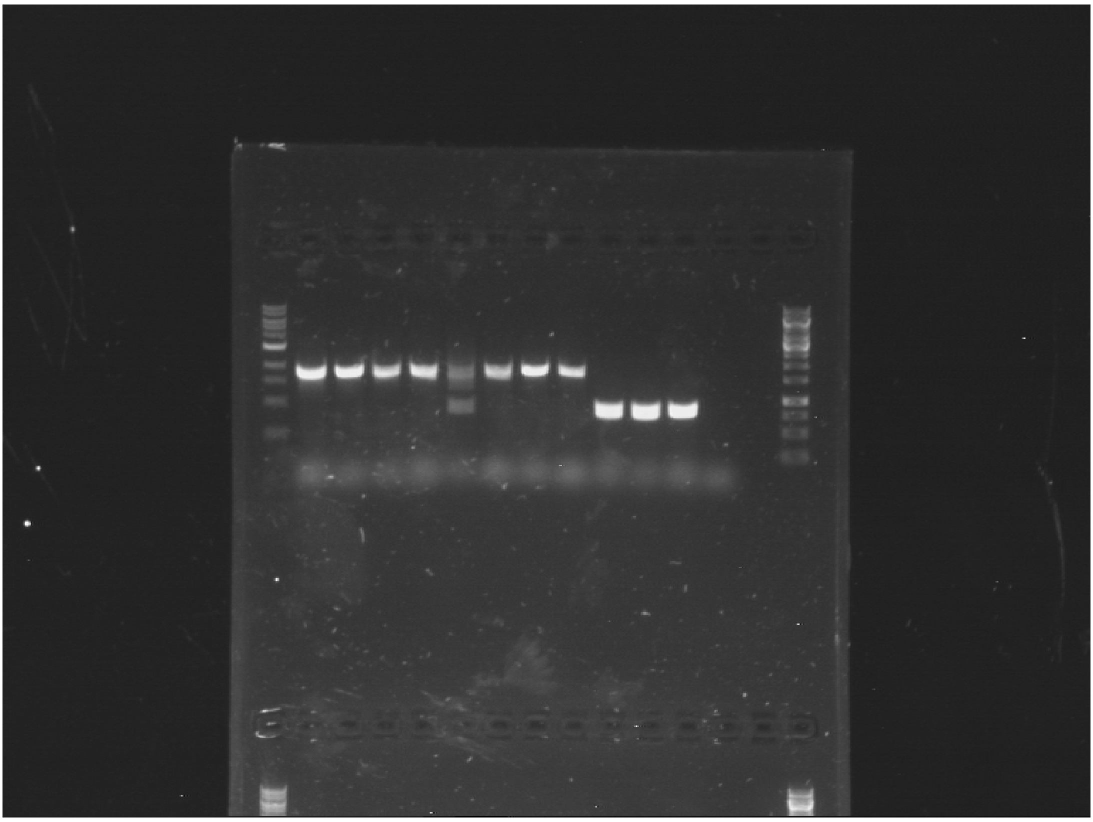

Uncropped gel image from Extended Data Figure 5B

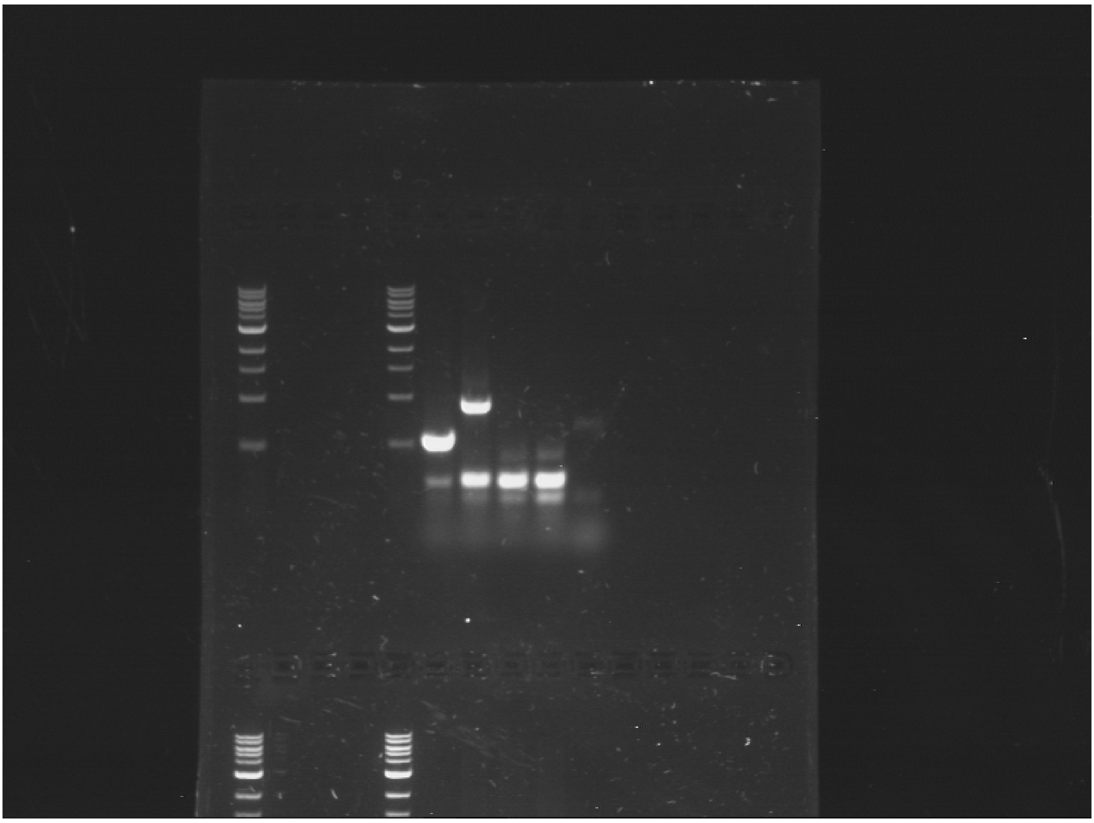

Supplement: Supplementary file 5 — Uncropped gels. [file 41564_2025_2182_MOESM5_ESM.pdf]
